# Spontaneous enanti­omorphism in poly-phased alkaline salts of tris­(oxalato)ferrate(III): crystal structure of cubic NaRb_5_[Fe(C_2_O_4_)_3_]_2_

**DOI:** 10.1107/S2056989018008022

**Published:** 2018-06-08

**Authors:** O. E. Piro, G. A. Echeverría, E. J. Baran

**Affiliations:** a Departamento de Física, Facultad de Ciencias Exactas, Universidad Nacional de La Plata and IFLP(CONICET), C.C. 67, 1900 La Plata, Argentina; b Centro de Química Inorgánica (CEQUINOR), Facultad de Ciencias Exactas, Universidad Nacional de La Plata, C.C. 962, 1900 La Plata, Argentina

**Keywords:** absolute crystal structures, spontaneous resolution of enanti­omorphs, sodium and rubidium salt of tris­(oxalato)ferrate(III)

## Abstract

We show here that the phenomenon of spontaneous resolution of enanti­omers occurs during the crystallization of the sodium and rubidium double salts of the transition metal complex tris­(oxalato)ferrate(III), namely sodium penta­rubidium bis­[tris­(oxalato)ferrate(III)], NaRb_5_[Fe(C_2_O_4_)_3_]_2_. One enanti­omer of the salt crystallizes in the cubic space group *P*4_3_32 with *Z* = 4 and a Flack absolute structure parameter *x* = −0.01 (1) and its chiral counterpart in the space group *P*4_1_32 with *x* = −0.00 (1). All metal ions are at crystallographic special positions: the iron(III) ion is on a threefold axis, coordinated by three oxalate dianions in a propeller-like conformation. One of the two independent rubidium ions is on a twofold axis in an eightfold coordination with neighbouring oxalate oxygen atoms, and the other one on a threefold axis in a sixfold RbO_6_ coordination. The sodium ion is at a site of *D*_3_ point group symmetry in a trigonal–anti­prismatic NaO_6_ coordination.

## Abstract 2

The phenomenon of spontaneous resolution of enanti­omers occurs during the crystallization of the sodium and rubidium double salts of the transition metal complex tris­(oxalato)ferrate(III).

## Chemical context   

Chirality is the structural property by which a mol­ecule or ion cannot be superposed upon its mirror image through translation and proper rotation operations. This concept along with the related ones of chiral crystal structures and space groups is discussed by Flack (2003 ▸). Chirality is at the core (among other research areas) of the not yet understood origin of the biomolecular asymmetry of life (Meierhenrich, 2008 ▸), enanti­oselective chemical reactions (Knowles, 2001 ▸; Noyori, 2001 ▸; Sharpless, 2001 ▸), biological activity of pharmaceuticals (Nguyen *et al.*, 2006 ▸) and in the design of multifunctional solid-state materials endowed with optical activity and long-range magnetic order (Coronado *et al.*, 2003 ▸) and also in the understanding of the physical properties of chiral liquid crystals and their tailoring for applications in opto-electronic devices (Goodby, 1998 ▸; Coles, 1998 ▸).

While attempting to crystallize the rubidium salt of the tris­(oxalato)ferrate(III) transition metal complex, one of the preparations segregated into a poly-phased crystal system. It contained the intended Rb_3_[Fe(C_2_O_4_)_3_]·3H_2_O compound (monoclinic *P*2_1_/*c*), which turned out to be isotypic to the reported potassium salt (Junk, 2005 ▸; Piro *et al.*, 2016 ▸), and the triclinic (*P* 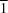) Rb(C_2_O_4_H)(C_2_O_4_H_2_)·2H_2_O salt (Kherfi *et al.*, 2010 ▸), which is isotypic to the ammonium analogue (Jarzembska *et al.*, 2014 ▸). A third phase consisted of large green crystals of a new cubic (*P*4_3_32) NaRb_5_[Fe(C_2_O_4_)_3_]_2_ salt. Inter­estingly, the isotypic counterpart of this salt where rubidium is replaced by potassium has been reported by Wartchow (1997 ▸) to appear in a mixture with crystals of the monoclinic K_3_[Fe(C_2_O_4_)_3_]·3H_2_O salt, hence confirming the tendency of potassium and rubidium alkaline ions to form isotypic crystal analogues (Piro *et al.*, 2016 ▸). Curiously, in a previous work, Henneicke & Wartchow (1997 ▸) reported the chiral counterpart of the cubic NaK_5_[Fe(C_2_O_4_)_3_]_2_ salt, which crystallizes in the space group *P*4_1_32. This prompted us to search for the chiral rubidium analogue in the very same batch as the single-crystals that solved in the space group *P*4_3_32 NaRb_5_[Fe(C_2_O_4_)_3_]_2_. By chance, we picked a single crystal and submitted it to X-ray diffraction scrutiny to find that it now belonged to the chiral space group *P*4_1_32. This strongly suggests that the Na*M*_5_[Fe(C_2_O_4_)_3_]_2_ (*M =* K, Rb) crystal samples could be racemic conglomerates generated by spontaneous resolution, a rare event discovered by Louis Pasteur in 1848 (Pasteur, 1848*a* ▸,*b* ▸) in a famous experiment in which he hand-sorted the chirally resolved crystals of sodium ammonium tartrate tetra­hydrate on the basis of their observed morphology and then examined their respective solutions with a polarimeter to find opposite rotations of the plane of light polarization (Flack, 2009 ▸). Recently, we found that the phenomenon could also have occurred in isotypic [*M*(Lap)_2_]_*n*_ (*M* = Cd, Mn; HLap = 2-hy­droxy-3-(3-methyl-2-buten­yl)-1,4-naphto­quinone, C_15_H_14_O_3_) complexes whose enanti­omers crystallize in the tetra­gonal and enanti­omorphic space groups *P*4_3_2_1_2 and *P*4_1_2_1_2 (Farfán *et al.*, 2015 ▸).

## Structural commentary   

**Figure 1:**
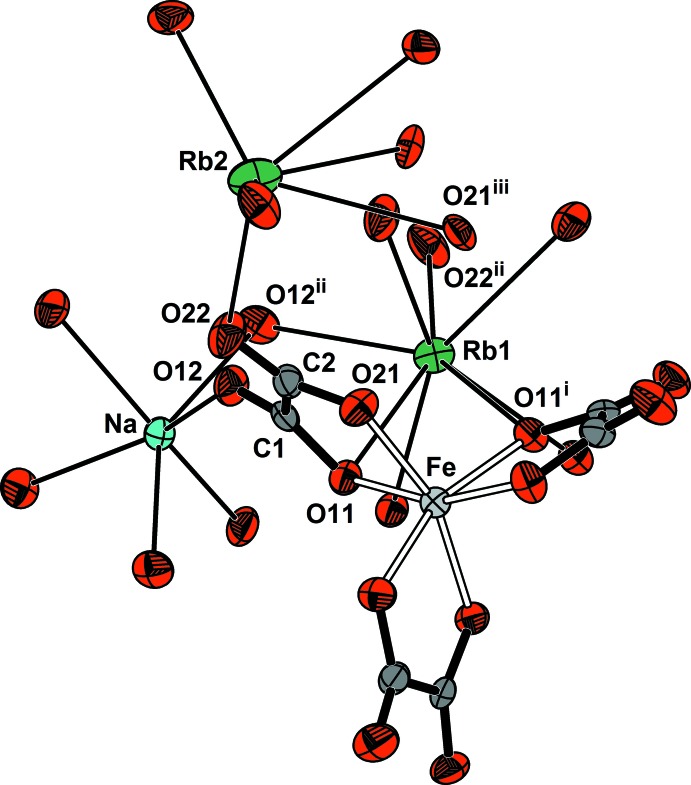
View of NaRb_5_[Fe(C_2_O_4_)_3_]_2_ showing the atom labels and displacement ellipsoids at the 50% probability level. For clarity, only the minimum number of oxygen ligands around each metal ion has been labelled. The rest of the environmental oxygen atoms are generated through the symmetry operations of the corresponding point groups: *C*_3_ (Fe), *C*_2_ (Rb1), *C*_3_ (Rb2) and *D*_3_ (Na). Iron–oxalate bonds are indicated by double lines and alkali metal–oxygen contacts by single lines. Symmetry codes: (i) −*y* + 1, *z* + 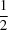, −*x* + 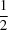; (ii) *y* − 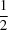, −*z* + 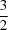, −*x* + 1; (iii) −*y* + 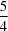, −*x* + 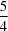, −*z* + 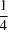.

Fig. 1 ▸ shows an *ORTEP* (Farrugia, 2012 ▸) drawing of the *P*4_3_32 enanti­omer of the title compound. Bond lengths and angles around iron(III) and within the oxalate dianion are listed in Table 1 ▸ and contact distances around the alkali ions are shown in Table 2 ▸. All metal ions are at crystallographic special positions while the oxalate anion is on a general position. The iron(III) ion is on a threefold axis, *C*_3_ point group symmetry (Wyckoft *c* site), in an octa­hedral environment (FeO_6_ core). It is coordinated to three, symmetry-related, oxalate anions acting as bidentate ligands through the oxygen atoms of their opposite carb­oxy­lic groups in a propeller-like conformation and along one electron pair lobe on each oxygen ligand. The FeO_6_ bond geometry and metrics are consistent with the oxalate being a weak-field ligand that gives rise to the high-spin (*S* = 5/2) electronic ground state exhibited by the complex, as probed by magnetic susceptibility (Delgado *et al.*, 2002 ▸) and ESR spectroscopy (Collison & Powell, 1990 ▸) in synthetic minguzzite, K_3_[Fe(C_2_O_4_)_3_]·3H_2_O, by polarized electronic absorption spectroscopy in single crystal NaMg[(Fe, Al)(C_2_O_4_)_3_]·9H_2_O mixtures (Piper & Carlin, 1961 ▸) and also by Mössbauer spectroscopy in K_3_[Fe(C_2_O_4_)_3_]·3H_2_O (Bancroft *et al.*, 1970 ▸; Sato & Tominaga, 1979 ▸; Ladriere, 1992 ▸) and in the alkali (Na, Rb, Cs) family of tris­(oxalato)ferrate(III) salts (Piro *et al.*, 2016 ▸).

The planes of the carb­oxy­lic –COO^−^ groups of the oxalate ligand are slightly tilted from each other, by 12 (1)° around the C—C σ-bond. As expected, the C—O bond lengths involving the coordinated-to-metal oxygen atoms are significantly longer [1.286 (7) and 1.283 (7) Å] than the ones corresponding to the uncoordinated oxalate oxygen atoms [both equal to 1.211 (7) Å].

There are two independent rubidium ions, one (Rb1) lying on a twofold axis, *C*_2_ point group symmetry (*d* site) in an eightfold coordination with neighbouring oxalate oxygen atoms, the other one (Rb2) on a threefold axis, *C*_3_ point group (*c* site) in a sixfold coordination. The sodium ion is at a site of *D*_3_ point group symmetry (*a* site) in a trigonal–anti­prismatic NaO_6_ coordination with one oxygen atom of six neighbouring, symmetry-related, oxalate ions.

**Figure 2:**
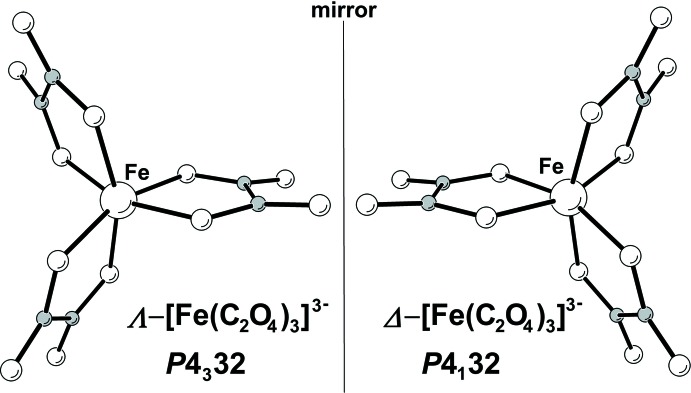
Views of the Λ and Δ enanti­omers of [Fe(C_2_O_4_)_3_]^3−^.

When dealing with octa­hedral Fe(C_2_O_4_)_3_ tris-chelated metal complexes, it is customary to describe its chirality employing Λ- and Δ-descriptors (Meierhenrich, 2008 ▸). It turns out that the enanti­omeric complexes correlate with the corresponding chiral space groups, as indicated in Fig. 2 ▸.

The possibility of controlling the crystal chirality and therefore obtaining enhanced optical activity of functional materials has been discussed (Gruselle *et al.*, 2006 ▸). To this purpose, two general synthetic routes have been developed to reach optically active coordination compounds, namely either by enanti­oselective synthesis using enanti­opure chiral species, which yields enanti­opure samples (Knof & von Zelewsky, 1999 ▸) or by spontaneous resolution upon crystallization from a racemate, which yields a conglomerate (Pérez-García & Amabilino, 2002 ▸). As explained above, the chiral NaRb_5_[Fe(C_2_O_4_)_3_]_2_ crystals were obtained through the phenomena of spontaneous resolution from a racemic solution of [Fe(C_2_O_4_)_3_]^3−^ complex ions into a racemic conglomerate. This is presumably followed by a structural inductive effect by these chiral mol­ecular ions on the alkali metal ions through shared oxalate ligands. In fact, not only is the Fe^III^ ion a ‘stereogenic centre’ in the Fe(C_2_O_4_)_3_ tris-chelated metal complex, but so also are the sodium and one (Rb2) of the rubidium ions. These metal ions are in a distorted octa­hedral environment coordinated to six oxalate anions, acting as monodentate ligand through one of their oxygen atoms and resembling a six-bladed propeller-like conformation. From the structural data, it turns out that the chirality of this local arrangement around the alkaline ions is coincident with the one of the [Fe(C_2_O_4_)_3_]^3−^ inductor and therefore the chiral crystals reported here can be more conveniently described as Λ-NaΛ-Rb_2_Rb_3_[Λ-Fe(C_2_O_4_)_3_]_2_ (*P*4_3_32) and Δ-NaΔ-Rb_2_Rb_3_[Δ-Fe(C_2_O_4_)_3_]_2_ (*P*4_1_32). However, no definitive chirality can be unambiguously assigned to the other independent rubidium (Rb1) ion which is in an eightfold polyhedral coordination.

## Database survey   

The formation of racemic conglomerates of single crystals, adequate for structural X-ray diffraction, generated by spontaneous resolution is an infrequent phenomenon. In fact, a search of the Cambridge Structural Database (Groom *et al.*, 2016 ▸) invoking the term ‘spontaneous resolution’ showed seventeen entries, and another one using as a target ‘chiral crystals’ produced a further four hits. Among them there were reported the chiral to each other (*M*)- and (*P*)-*catena*-{[μ_2_-2-(imidazo[4,5-*f*](1,10)phenanthrolin-2-yl)benzoato-*N*,*N*′,*O*]aqua­chloro­zinc(II)} (CSD refcodes EJINOB and EJINUH; Wei *et al.*, 2011 ▸) and *catena*-[(μ_8_-benzene-1,3,5-tri­carboxyl­ato)lithiumzinc] (CSD refcodes WAJHUM and WAJJAU; Xie *et al.*, 2010 ▸).

## Synthesis and crystallization   

As stated in the *Chemical context*, in one of the preparations generated during the synthesis of the rubidium salt of [Fe(C_2_O_4_)_3_]^3−^, by reaction of freshly precipitated Fe(OH)_3_ (obtained by dropwise addition of a small excess of 20% NaOH to an Fe^III^ solution) with rubidium oxalate: Fe(OH)_3_ + 3Rb(HC_2_O_4_) + 3H_2_O → Rb_3_[Fe(C_2_O_4_)_3_]·3H_2_O + 3H_2_O) (Piro *et al.*, 2016 ▸), we found a relatively complex reaction giving rise to a poly-phased crystal mixture, from which the NaRb_5_[Fe(C_2_O_4_)_3_]_2_ chiral pair could be isolated.

## Refinement details   

Crystal data, data collection procedure and structure refinement results are summarized in Table 3 ▸. The structure was solved by intrinsic phasing with *SHELXT* (Sheldrick, 2015*a* ▸). The stereoisomers were determined through refinement of the Flack absolute structure parameter. This is the fractional contribution to the diffraction pattern due to the mol­ecule racemic twin and for the correct enanti­omeric crystal it should be zero to within experimental error.

## Supplementary Material

Crystal structure: contains datablock(s) P4332, P4132, global. DOI: 10.1107/S2056989018008022/hb7735sup1.cif

Structure factors: contains datablock(s) P4332. DOI: 10.1107/S2056989018008022/hb7735P4332sup2.hkl

Structure factors: contains datablock(s) P4132. DOI: 10.1107/S2056989018008022/hb7735P4132sup3.hkl

CCDC references: 1563459, 1563458

Additional supporting information:  crystallographic information; 3D view; checkCIF report

## Figures and Tables

**Table 1:** Bond lengths and angles (Å, °) around iron(III) and within the oxalate dianion in NaRb_5_[Fe(C_2_O_4_)_3_]_2_ *P*4_3_32 enanti­omer (*a*) At a crystal site of *C*_3_ point group symmetry.

Iron(III)*^*a*^*		(C_2_O_4_)^2−^			
Fe—O11	2.021 (4)	C1–O12	1.211 (7)	O12—C—O11	125.2 (6)
Fe—O21	1.989 (4)	C1–O11	1.286 (7)	O12—C1—C2	121.2 (6)
		C1—C2	1.540 (9)	O11—C1—C2	113.5 (5)
O21—Fe—O11	80.0 (2)	C2—O22	1.211 (7)	O22—C2—O21	125.3 (6)
O21—Fe—O21^i^	88.4 (2)	C2—O21	1.283 (7)	O22—C2—C1	121.1 (6)
O11—Fe—O11^i^	88.7 (2)			O21—C2—C1	113.6 (5)
O11—Fe—O21^i^	106.2 (2)				
O11—Fe—O21^ii^	160.9 (2)				

Symmetry codes: (i) −*z* + 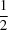, −*x* + 1, *y* − 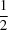; (ii) −*y* + 1, *z* + 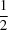, −*x* + 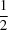.

**Table 2:** Bond lengths (Å) around the alkali metal ions in NaRb_5_[Fe(C_2_O_4_)_3_]_2_ *P*4_3_32 enanti­omer. (*a*) At a site of *C*_2_ point group symmetry; (*b*) at a *C*_3_ site; (*c*) at a *D*_3_ site.

Rb1*^*a*^*		Rb2*^*b*^*		Na^*c*^	
Rb1—O11	3.009 (4)	Rb2—O22	2.808 (4)	Na—O12	2.439 (4)
Rb1—O11^i^	3.067 (4)	Rb2—O21^iii^	3.114 (4)		
Rb1—O22^ii^	2.788 (5)				
Rb1—O12^ii^	3.133 (5)				

Symmetry codes: (i) −*y* + 1, *z* + 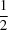, −*x* + 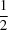; (ii) *y* − 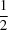, −*z* + 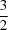, −*x* + 1; (iii) −*y* + 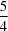, −*x* + 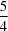, −*z* + 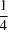.

**Table 3:** Experimental details

	Cubic, *P*4_3_32	Cubic, *P*4_1_32
Crystal data		
Chemical formula	NaRb_5_[Fe(C_2_O_4_)_3_]_2_	NaRb_5_[Fe(C_2_O_4_)_3_]_2_
*M*_r_	1090.16	1090.16
Temperature (K)	297	293
*a* (Å)	13.8058 (4)	13.7995 (3)
*V* (Å^3^)	2631.4 (2)	2627.79 (17)
*Z*	4	4
Radiation type	Mo *K*α	Mo *K*α
μ (mm^−1^)	10.42	10.43
Crystal size (mm)	0.48 × 0.42 × 0.38	0.48 × 0.35 × 0.25

Data collection		
Diffractometer	Agilent Xcalibur Eos Gemini	Rigaku Oxford Diffraction Xcalibur, Eos, Gemini
Absorption correction	Multi-scan (*CrysAlis PRO*; Agilent, 2014 ▸)	Multi-scan (*CrysAlis PRO*; Rigaku OD, 2015 ▸)
*T*_min_, *T*_max_	0.690, 1.000	0.786, 1.000
No. of measured, independent and observed [*I* > 2σ(*I*)] reflections	2960, 959, 767	4284, 961, 814
*R*_int_	0.043	0.038
(sin θ/λ)_max_ (Å^−1^)	0.638	0.638

Refinement		
*R*[*F* ^2^ > 2σ(*F* ^2^)], *wR*(*F* ^2^), *S*	0.035, 0.064, 1.00	0.032, 0.068, 1.02
No. of reflections	959	961
No. of parameters	68	68
Δρ_max_, Δρ_min_ (e Å^−3^)	0.84, −0.85	1.02, −0.95
Absolute structure	Flack *x* determined using 225 quotients [(*I* ^+^)−(*I* ^−^)]/[(*I* ^+^)+(*I* ^−^)] (Parsons *et al.*, 2013 ▸)	Flack *x* determined using 251 quotients [(*I* ^+^)−(*I* ^−^)]/[(*I* ^+^)+(*I* ^−^)] (Parsons *et al.*, 2013 ▸).
Absolute structure parameter	−0.013 (12)	−0.003 (10)

Computer programs: *CrysAlis PRO* (Agilent, 2014 ▸; Rigaku OD, 2015 ▸), *SHELXT* (Sheldrick, 2015*a* ▸), *SHELXL2014* (Sheldrick, 2015*b* ▸) and *ORTEP-3 for Windows* (Farrugia, 2012 ▸).
